# The Use of OMT in the Emergency Department: A Scoping Review

**DOI:** 10.51894/001c.144112

**Published:** 2025-09-30

**Authors:** Peter Nemes

**Affiliations:** 1 College of Osteopathic Medicine Michigan State University

## 33

### INTRODUCTION

Osteopathic Manipulative Treatment (OMT) has been utilized to treat a variety of conditions in patients of all ages in both the inpatient and outpatient setting. Musculoskeletal complaints are a common reason for patients presenting to the emergency department (ED), yet the application of OMT in the emergency department has not been well established.

### OBJECTIVES

The purpose of this scoping review was to assess the use of OMT in the ED, particularly the frequency of use, techniques utilized, and conditions commonly treated.

### METHDOS

The scoping review process was conducted in accordance with the guidelines established by the Preferred Reporting Items for Systematic Reviews and Meta-Analyses extension for Scoping Reviews (PRISMA-ScR). The literature search was conducted using PubMed, CINAHL, Embase, and Scopus. All peer-reviewed publications were considered. Two reviewers independently screened the records, and a third reviewer resolved any disagreements and facilitated consensus. Data extraction was performed using a standardized data extraction form, followed by a descriptive synthesis to summarize the data.

### RESULTS

A total of 283 publications were identified across all databases. After removing duplicates and applying the inclusion criteria, a total of 8 studies were included. These studies were comprised of case reports (n=4), retrospective reviews (n=2), and randomized controlled trials (n=2), with a total of 2,194 participants. In the ED setting, OMT was used to treat patients with neck pain, low back pain, muscle spasms, foot and ankle sprains, rib dysfunction, abdominal pain, migraines, and singultus. OMT techniques included muscle energy technique, myofascial release, osteopathic cranial manipulation, high-velocity low-amplitude, balanced ligamentous tension, fascial distortion model, soft tissue techniques, Still technique, and counterstrain, though the frequency was not consistently reported.

### CONCLUSIONS

OMT can be a viable treatment option for a variety of conditions in the ED. The literature regarding its use in the ED is limited in both the number of articles and size of studies. Further research is needed to demonstrate the efficacy and applicability of OMT in the ED.

